# A Novel Salivary Sensor with Integrated Au Electrodes and Conductivity Meters for Screening of Diabetes

**DOI:** 10.3390/bios13070702

**Published:** 2023-07-02

**Authors:** Chen-Wei Lin, Yuan-Hsiung Tsai, Yun-Shing Peng, Jen-Tsung Yang, Yen-Pei Lu, Mei-Yen Chen, Chun-Wu Tung

**Affiliations:** 1 Department of Medical Education, Chang Gung Memorial Hospital, Chiayi 61363, Taiwan; toddgod7@cgmh.org.tw; 2 Department of Diagnostic Radiology, Chang Gung Memorial Hospital, Chiayi 61363, Taiwan; 3 College of Medicine, Chang Gung University, Taoyuan 33302, Taiwan; 4 Division of Endocrinology and Metabolism, Department of Internal Medicine, Chang Gung Memorial Hospital, Chiayi 61363, Taiwan; 5 Department of Neurosurgery, Chang Gung Memorial Hospital, Chiayi 61363, Taiwan; 6 National Applied Research Laboratories, Taiwan Instrument Research Institute, Hsinchu 30261, Taiwan; 7 Department of Nursing, Chang Gung University of Science and Technology, Chiayi 61363, Taiwan; 8 Department of Nephrology, Chang Gung Memorial Hospital, Chiayi 61363, Taiwan; 9 Chang Gung Medical Education Research Centre, Taoyuan 33302, Taiwan; 10 Department of Biochemical Science and Technology, National Chiayi University, Chiayi 61363, Taiwan

**Keywords:** diabetes mellitus, salivary conductivity, sensor, non-invasive, blood sugar self-monitoring

## Abstract

The rise in diabetes cases is a growing concern due to the aging of populations. This not only places a strain on healthcare systems but also creates serious public health problems. Traditional blood tests are currently used to check blood sugar levels, but they are invasive and can discourage patients from regularly monitoring their levels. We recently developed nano-sensing probes that integrate Au microelectrodes and conductivity meters, requiring only 50 μL of saliva for measurement. The usage of the co-planar design of coating-free Au electrodes makes the measurement more stable, precise, and easier. This study found a positive correlation between the participant’s fasting blood sugar levels and salivary conductivity. We observed a diabetes prevalence of 11.6% among 395 adults under 65 years in this study, using the glycated hemoglobin > 6.5% definition. This study found significantly higher salivary conductivity in the diabetes group, and also a clear trend of increasing diabetes as conductivity levels rose. The prediction model, using salivary conductivity, age, and body mass index, performed well in diagnosing diabetes, with a ROC curve area of 0.75. The study participants were further divided into low and high groups based on salivary conductivity using the Youden index with a cutoff value of 5.987 ms/cm. Individuals with higher salivary conductivity had a 3.82 times greater risk of diabetes than those with lower levels, as determined by the odds ratio calculation. In conclusion, this portable sensing device for salivary conductivity has the potential to be a screening tool for detecting diabetes.

## 1. Introduction

Diabetes is a major chronic disease that not only poses significant public health problems but also results in significant economic losses [1,2,3,4]. Currently, it is estimated to affect approximately 10.5% of the world’s population, and its prevalence is on the rise [5]. Metabolic syndrome is a serious complication of diabetes, but the prolonged state of abnormal blood sugar is also associated with several other comorbidities [6]. Diabetes patients are also more vulnerable to other illnesses, placing them at higher risk of prolonged hospitalization, greater mortality rate, and reduced quality of life [7]. The current standard for diagnosing diabetes and monitoring blood glucose levels involves a blood test, which is invasive and may cause short-term complications, such as pain or local infections [8,9,10]. Therefore, a rapid, non-invasive, and user-friendly method for measuring blood glucose levels is necessary to enhance patient engagement in monitoring their health.

Saliva is an excellent bodily fluid specimen for health evaluation because it can be obtained non-invasively and contains a diverse array of water, electrolytes, cytokines, antibodies, and metabolites [11,12,13,14,15]. Saliva is a valuable biomarker for monitoring different diseases, and its collection is simple and inexpensive [16,17,18]. While some studies have shown a correlation between salivary and blood glucose levels, measuring it requires sophisticated instruments [19,20,21]. Our research team is dedicated to creating a portable and easy-to-use saliva-analyzing device to investigate its potential as a diagnostic biomarker for diabetes.

Salivary electrolyte concentrations, such as sodium, potassium, calcium, chloride, and bicarbonate, significantly increase with the advancement of diabetes [22,23,24,25]. It is believed that changes in the permeability of the salivary gland and blood vessel basement membranes caused by prolonged hyperglycemia can lead to significant increases in the salivary electrolytes. Additionally, patients with diabetes often experience xerostomia due to low saliva secretion rates, which can alter the electrical conductivity of saliva [24,25]. Our team has developed a novel sensing device with miniaturized probes for measuring salivary conductivity [26]. The portable device is easy-to-use and does not require specialized personnel to operate. Our initial data suggests a positive correlation between higher salivary conductivity and elevated fasting glucose levels, as well as an increased risk of developing chronic kidney disease [27,28,29]. However, further research is necessary to validate salivary conductivity as a reliable biomarker for diabetes. Therefore, this pilot study aims to confirm the association between salivary conductivity and diabetes in healthy adults and explore the potential of salivary conductivity as a screening tool for diabetes.

## 2. Materials and Methods

### 2.1. The Sensing Device

**Figure 1:**
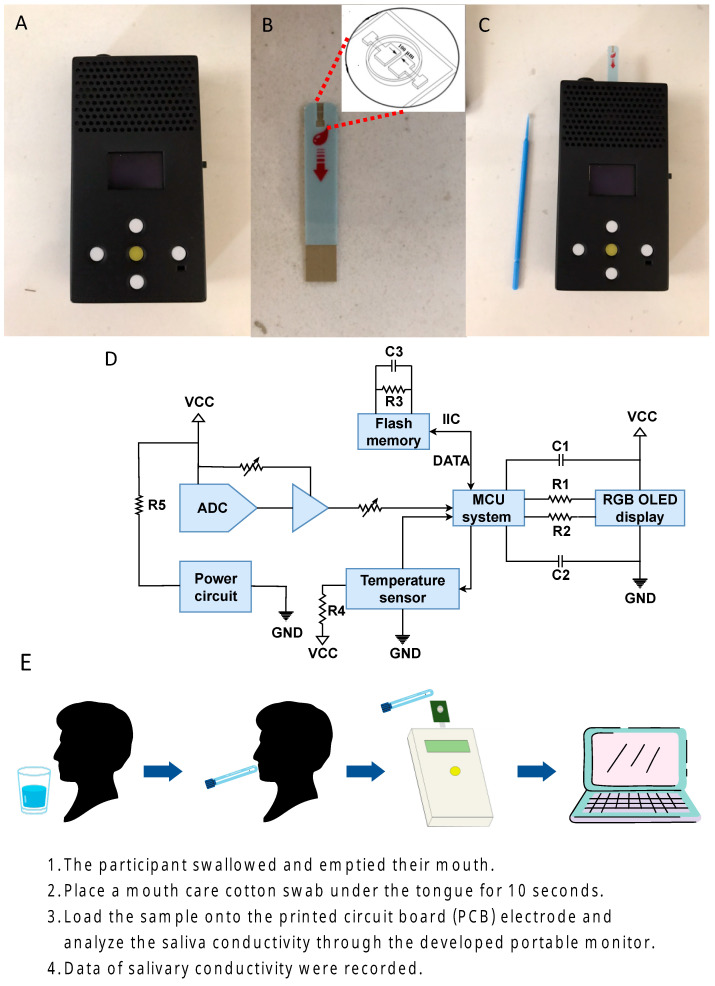
The portable device and the principles for measuring salivary conductivity. (**A**) This portable sensing system has dimensions of 10 × 5.5 × 2.2 cm^3^. (**B**) A PCB equipped with co-planar electroless nickel immersion gold electrodes that are free of coating and located in the sample well. (**C**) Connection of the two components and the saliva sample collection swab. (**D**) Electronic schematics of the fabricated conductivity meter. (**E**) Steps for saliva collection and measurement of conductivity. Abbreviations: ADC, analog-to-digital converter; (**C**) capacitance; GND, ground; IIC, inter-integrated circuit; MCU, micro control unit; OLED, organic light-emitting diode; PCB, printed-circuit-board; R, resistance; RGB, red, green, blue color model; VCC, volt current condenser.

A portable device has been developed for determining the conductivity of saliva. It comprises two components, a conductivity meter, and a printed circuit board (PCB) equipped with electroless nickel immersion gold electrodes to prevent the oxide layer over the probe (shown in Figure 1A–C). The micro-fabricated gold electrodes are 2 × 2 mm^2^ in size and connected to the PCB using nickel immersion gold wire. The compact size of the electrodes means that only 50 μL of the test sample is needed to cover the electrodes adequately (as illustrated in Figure 1B). The conductivity meter, measuring 10 × 5.5 × 2.2 cm^3^, includes a temperature sensor from Aosong (Guangzhou, China), a micro-control unit (MCU system) from STMicroelectronics (Geneva, Switzerland), an analog-to-digital converter (ADC) from Analog Devices (Norwood, MA, USA), and an organic light-emitting diode (OLED) from Zhongjingyuan (Henan, China). The conductivity meter can be activated using the ADC with 1 App and 1 kHz sine waves, and the conductivity parameters can be computed through a discrete Fourier transform. The temperature compensation through the MCU system allows the measurement signal to be obtained at 25 °C, and the result can be displayed on the OLED within 10 s (Figure 1D). Our device is highly reusable, as supported by previous research [29].

### 2.2. Saliva Collection and Analysis

The collection and analysis of saliva samples follow established protocols from prior research [27,28,29]. In brief, the process involves four key steps (Figure 1E). Firstly, participants are instructed to rinse their mouths with tap water for 30 s and empty any excess saliva. Secondly, they hold a saliva sample collection swab under their tongue for 10 s to collect saliva. Thirdly, the swab is inserted into the sample well of the sensing probe for analysis. Lastly, the conductivity is measured and displayed on the conductivity meter. The entire process, including preparation, takes approximately 3 min to complete.

### 2.3. Study Participants

**Figure 2:**
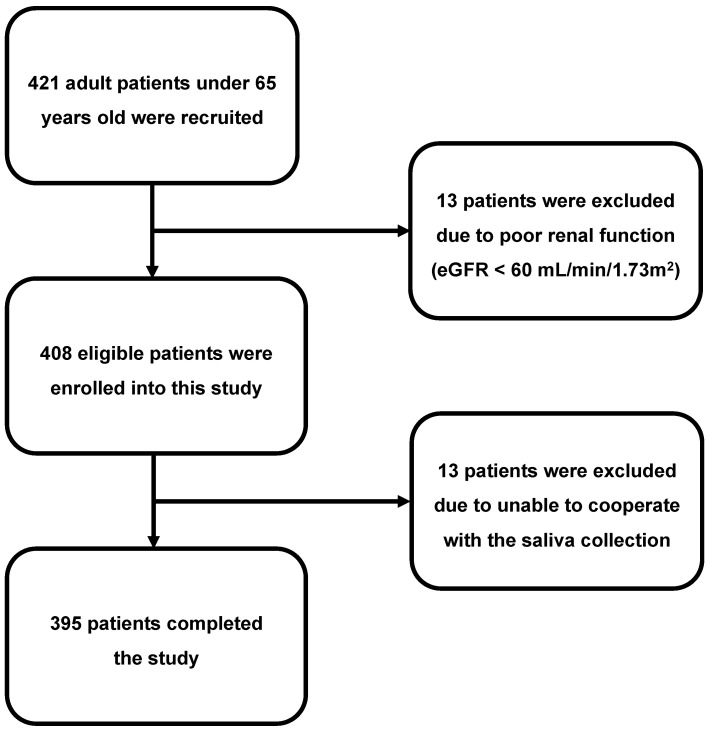
Flow chart of patient enrollment. The diagram illustrates the enrollment status of the participants.

This pilot study, with a cross-sectional design, enrolled adults aged between 18 and 65 years. A total of 421 subjects were recruited during their annual health check-up at the Yunlin Branch of the Chang Gung Memorial Hospital, a regional teaching hospital located in central Taiwan, in August 2021. After excluding participants with poor renal function, 408 eligible subjects were enrolled, but 13 of them were later excluded due to difficulties in cooperating with the saliva collection process, such as rinsing the mouth or placing cotton swabs under the tongue. Ultimately, this study was completed by 395 adult participants (Figure 2). This study adhered to the guidelines of the Declaration of Helsinki and was approved by the Medical Ethics Committee of Chang Gung Memorial Hospital (institutional review board numbers: 202002186B0). All participants provided informed consent before participating in this study.

### 2.4. Clinical Study Design

Before their health examination, participants were asked to fill out a comprehensive questionnaire provided by trained nurses. The questionnaire covered basic health information, including medical histories, such as diabetes, chronic kidney disease, ischemic heart disease, stroke, dyslipidemia, hypertension, gout, and cancer. Body height, weight, and corresponding body mass index (BMI) were measured, and blood pressure was taken in a quiet environment. Participants were instructed to fast overnight before the examination. Both blood and saliva samples were collected and analyzed. The blood samples were processed using an automatic chemistry analyzer (Beckman DXC880i, Brea, CA, USA) following established laboratory procedures. The saliva samples were collected twice to calculate the average of the salivary conductivity. If the results of the two tests were inconsistent, the collection steps would be repeated to make sure the saliva samples were collected in the right way. Based on our previous work, we have shown that our device can yield great reusability, with the mean absolute percentage error (MAPE) being 0.88% after measuring the same saliva sample 20 times [29].

### 2.5. Definition of Diabetes

Diabetes is defined as having a glycated hemoglobin A1c (HbA1c) level higher than 6.5%, according to the recommendation of the American Diabetes Association.

### 2.6. Statistical Analysis

In this study, continuous variables are expressed as means with standard deviations, while categorical variables are presented as the number and percentage of observations. For comparing two groups, the independent Student’s *t*-test was used for continuous variables with a normal distribution, and the Mann–Whitney U test was used for continuous variables that were not normally distributed. The normality of numerical variables was assessed using the Kolmogorov–Smirnov method. The results of the normality tests and the distribution plots of the salivary conductivity among different groups were shown in the Supplementary materials (Tables S1 and S2, Figure S1). Pearson’s chi-square test was employed for comparing multiple groups of categorical variables. To evaluate the diagnostic accuracy of salivary conductivity in diabetes, receiver operating characteristic (ROC) curve analysis was used, and multivariable logistic regression analysis with backward selection was applied to improve its diagnostic performance. The diagnostic power was determined by calculating the area under the ROC curve (AUROC). The null hypothesis was rejected at a 95% confidence interval, and all statistical analyses were two-sided. The statistical analysis was performed using the Statistical Program for Social Sciences (SPSS) version 26 (IBM Corporation, Armonk, NY, USA) and Python version 3.10.

## 3. Results

### 3.1. Demographic Characteristics of the Study Group

The results of this study showed that the mean age of the participants was 51.78 years with a standard deviation of 11.31 years, and the age ranged from 15 to 65 years old. Of the total participants, 126 (31.9%) were male, while the rest were female. The survey revealed that 46 participants (11.6%) had diabetes, 95 (24.1%) had hypertension, 47 (11.9%) had dyslipidemia, and 11 (2.8%) had gout. The mean fasting glucose and hemoglobin A1c levels were 108.40 mg/dL and 5.88%, respectively. The mean value of salivary conductivity was 5.58 ms/cm. The remaining anthropometric parameters, blood pressure, and biochemical data are presented in Table 1.

### 3.2. Comparison of DM versus Non-DM Study Group

The participants were divided into the DM (Diabetes mellitus) and non-DM groups based on their HbA1c levels. DM group was defined by an HbA1c level greater than 6.5%, while the non-DM group was the rest of the participants. In the DM group (*n* = 46), the mean salivary conductivity value was 6.29 ± 1.58 ms/cm, which was higher than that in the non-DM group (5.48 ± 1.59 ms/cm, *n* = 349). Additionally, the DM group had significantly higher age and BMI, and a higher prevalence of comorbidities such as hypertension and dyslipidemia than the non-DM group. Furthermore, the DM group had significantly higher serum osmolarity and fasting glucose levels compared to the non-DM group. Hemoglobin A1c levels were also significantly higher in the DM group (7.80 ± 1.51%) than in the non-DM group (5.63 ± 0.35) (Table 1). To minimize any potential bias that may have resulted from the unequal distribution of confounding variables, we utilized subgroup analysis to compare the male and female groups (Tables S3 and S4) and propensity score matching analysis (Table S5). These results were consistent with the initial analysis that gender or other demographic data would not be the confounding factors. It suggests that the salivary conductivity value could serve as a potential biomarker for diabetes mellitus.

### 3.3. Relationship between Salivary Conductivity and Diabetes Mellitus

**Figure 3:**
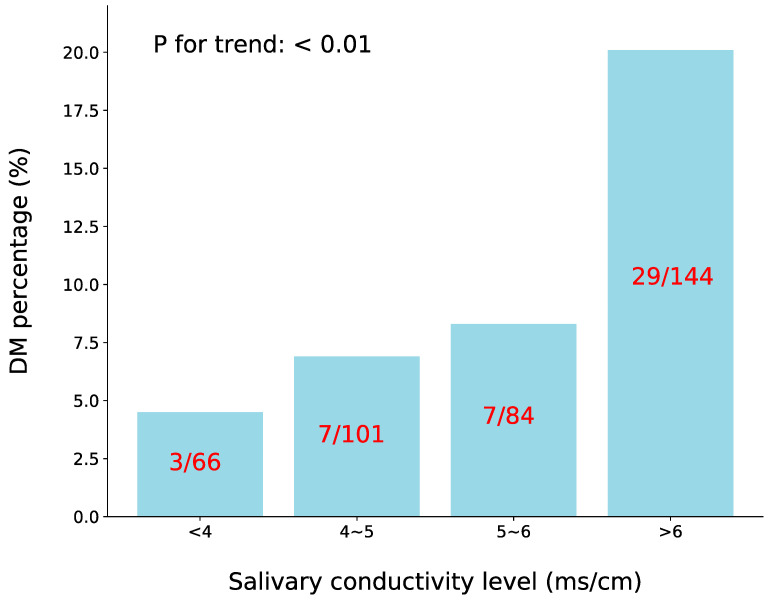
A bar chart showing the percentage of participants with DM at different levels of salivary conductivity. The number of subjects with DM and each group is also displayed in the graph (*p* for trend < 0.01). DM, diabetes mellitus.

This study then evaluated the association between salivary conductivity and diabetes by categorizing the participants into four groups according to their salivary conductivity levels (Figure 3). As the salivary conductivity levels increased, the incidence of diabetes also increased, with diabetes prevalence being 4.5%, 6.9%, 8.3%, and 20.1% for the four groups, respectively (with a *p*-value for the trend < 0.01).

### 3.4. The Use of Salivary Conductivity to Diagnose Participants with Diabetes Mellitus

**Figure 4:**
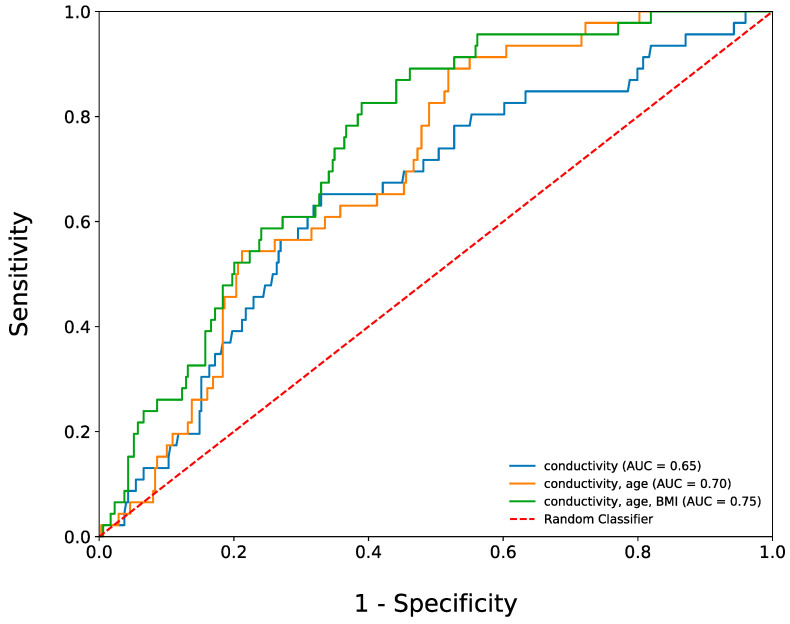
Three receiver operating characteristic curves. The AUROC is equal to 0.65 when using salivary conductivity as the only predicting factor. When combing salivary conductivity, age, and BMI as the predicting factors, the AUROC can be increased to 0.75. AUROC, the area under the receiver operating characteristic curve.

We further assessed the diagnostic ability of salivary conductivity in detecting diabetes by performing a ROC curve analysis. Using only salivary conductivity as the predictor, the AUROC was 0.654 (95% CI: 0.563–0.744). To enhance the predictive performance of the model, we incorporated age and BMI as additional predictors since they can be measured non-invasively. The AUROC increased to 0.698 (95% CI: 0.610–0.787) when salivary conductivity and age were combined as predictors. When BMI was also included, the AUROC further increased to 0.749 (95% CI: 0.664–0.833) (Figure 4).

### 3.5. Comparison of Low versus High Salivary Conductivity Study Group

The study participants were further divided into two groups based on their salivary conductivity levels, using a cutoff value (5.987 ms/cm) determined by the Youden index of the ROC curve. The low group had a mean salivary conductivity level of 4.57 ± 0.81 ms/cm, while the high group had a mean of 7.33 ± 1.06 ms/cm. Table 2 shows a comparison of different variables between the two groups. The results showed that participants in the low salivary conductivity group were younger and had lower body weight, BMI, systolic and diastolic blood pressure, and a lower likelihood of having diabetes, hypertension, and dyslipidemia, in comparison to those in the high salivary conductivity group. Additionally, they had lower levels of BUN, creatinine, fasting glucose, and HbA1c and higher levels of eGFR than those in the high group.

### 3.6. Associations between Salivary Conductivity and the Risk of Diabetes

The odds of having diabetes were determined for participants with high and low salivary conductivity. The crude odds ratio was 3.82 (95% CI: 1.44–5.56), indicating that subjects with higher salivary conductivity were 3.82 times more likely to have diabetes compared to those with lower conductivity. The adjusted odds ratio, calculated using two different multivariate models, was 3.35 and 2.69, respectively (Table 3). Table 4 displays the crude and adjusted odds ratios for other clinical parameters.

## 4. Discussion

The presented results indicate a positive correlation between salivary conductivity and HbA1c and fasting glucose, implying that salivary conductivity may serve as a potential biomarker for detecting diabetes and monitoring blood sugar levels. Blood tests for glucose or HbA1c are typically regarded as the gold standard for diabetes diagnosis. However, such tests are invasive and may involve procedures such as venipuncture and fingertip pricking, which patients often find burdensome and are, therefore, less willing to perform regularly. In our previous research, a strong positive correlation between salivary conductivity and fasting glucose or HbA1c was found through Pearson’s correlation test [29]. Additionally, as salivary conductivity levels increased, so did the prevalence of diabetes. Thus, salivary conductivity may offer a promising alternative to traditional methods of monitoring blood sugar levels.

In our study, we found that the prevalence rate of diabetes, as defined by the American Diabetes Association, was 11.6%. This is consistent with the national prevalence rate of 10.10% reported in a large-scale cohort study conducted in Taiwan in 2014, suggesting that our study participants can be considered representative of the general population [30]. Our previous study also showed a positive correlation between salivary conductivity and age or creatinine, and a negative correlation with eGFR [27,28]. These findings align with those of the current study. Participants with high salivary conductivity were older, had higher fasting sugar and HbA1c levels, and were at a higher risk of developing chronic kidney disease. It is known that the properties and secretion of saliva change with age, leading to an increase in electrolytes, as well as a direct link between salivary conductivity and age [28,31,32,33,34]. Furthermore, serum glucose levels have been shown to directly reflect salivary glucose concentration, which supports the use of saliva as a predictor of blood sugar levels [35]. Salivary conductivity was also found to be positively correlated with body weight, BMI, and blood pressure. Given that diabetes patients are often overweight, this may partly explain why they have higher salivary conductivity levels [23].

We have developed a sensor that is utilized for measuring salivary conductivity, which is determined by the electrical admittance between the electrodes. This measurement predominantly reflects the concentration of electrolytes. To enhance selectivity, we have implemented specific design features in our device. Firstly, considering that the signal follows the path of least resistance, electroimpedance spectroscopy primarily occurs at the periphery of the microelectrodes. As a result, interference effects caused by larger particles like food debris and nasal secretion, which typically settle on the top surface of the co-planar electrode, are minimized, leading to improved selectivity. Secondly, to evaluate the stability of the saliva sample, we conducted additional experiments since interfering particles may accumulate over time on the surface of the salivary solution. The findings indicated that the saliva sample exhibited good stability over a prolonged duration. Thirdly, a mere 50 μL saliva sample is required for conducting the test. Consequently, we can assume that the sample temperature quickly reaches equilibrium with the surrounding ambient temperature.

The ROC curve analysis of our study indicated that salivary conductivity, in combination with age and BMI, can be used as a good predictive model with an AUROC of 0.75. Although body weight and BMI can both serve as predictive factors, we selected BMI as it provides more information about an individual’s body shape. Our model’s sensitivity and specificity were not as good as those of traditional blood glucose meters, but it still represents a useful tool for self-monitoring sugar levels. Pain is a significant obstacle to self-monitoring blood glucose among patients with type 2 diabetes [10], and a device that can indirectly measure blood sugar levels non-invasively, even if less accurately, may be more useful than a device that is more accurate but also invasive if patients need to check their blood sugar multiple times per day.

According to a review article written by Tang et al., they have divided non-invasive blood glucose monitoring technology into three categories: optics, microwave, and electrochemistry [21]. The optics method can be subdivided into five categories, including near-infrared spectroscopy, optical polarimetry, Raman spectroscopy, fluorescence method, and optical coherence tomography. The electrochemistry method can be subdivided into reverse iontophoresis technology and non-invasive biofluid-based glucose monitoring. The advantages of optics and microwave methods are that they are more non-invasive and provide the possibility to continuous monitoring blood sugar levels, while less accuracy and poor correlation to actual blood glucose are the disadvantages. The electrochemistry method can predict more accurate blood sugar levels but delay in measurement results, the need for calibration, and biofluid collection are the defects. A meta-analysis by Lindner et al. has proposed that the diagnostic accuracy of non-invasive glucose monitoring devices is still not sufficiently accurate for blood sugar monitoring due to low sensitivity [36]. Although our prediction model result is similar to the previous work, we still provided a different and potential idea for monitoring blood sugar non-invasively. In addition, the same method has been proposed for detecting chronic kidney disease in our previous article [29]. It means that saliva could indeed reflect the physiological change in our body.

Localized surface plasmon resonance (LSPR) technology is a promising label-free biosensing technique. It utilizes the sensitivity of the plasmon frequency to changes in the local index of refraction at the nanoparticle surface. It has been used to detect several biomolecules, such as creatinine, troponin I, and aflatoxin [37,38,39]. Using LSPR technology to detect blood sugar is under investigation but still lacks robust evidence. Therefore, combining our salivary conductivity meter with the LSPR technology could be a possible method to increase accuracy when monitoring blood glucose levels in the future. 

There are several limitations to this study. Firstly, it is a cross-sectional study, and we did not collect longitudinal data on each participant’s blood glucose level, so we cannot definitively confirm the association between salivary conductivity and blood sugar levels. Secondly, we did not distinguish between type 1 and type 2 diabetes, although the prevalence of type 1 diabetes among mostly adult participants is likely to be very low. Thirdly, diabetes was defined as HbA1c higher than 6.5%, which is a narrower definition than the gold standard and may underestimate the prevalence of diabetes. However, our study results, both from the questionnaire survey and blood test, align with the national prevalence rate in Taiwan, suggesting that our study participants are still representative. Fourth, the study participants were collected during an annual health examination, which might attract individuals who are more health conscious. This could limit the generalizability of the findings to the broader population. Fifth, the saliva was collected when the patients were mildly dehydrated in the morning. We did not compare the saliva collected from different instances in which the flow rate or composition of the saliva may alter. Finally, we did not analyze the components of saliva, such as electrolytes, in the diabetes versus the normal group, so we were unable to clearly compare the underlying reason for their difference in salivary conductivity and justify our mechanism hypothesis. Further studies should focus on the limitations mentioned above to enhance the robustness and generalizability of the findings, strengthen the relationship between salivary conductivity and diabetes, and prove that salivary conductivity can be a reliable biomarker to monitor blood glucose levels.

## 5. Conclusions

Regular monitoring of blood glucose and prompt diagnosis of diabetes is crucial in reducing the associated health risks. The findings of this study demonstrate the relationship between salivary conductivity and blood sugar levels, with higher salivary conductivity associated with higher fasting glucose or HbA1c levels. Our research also shows that using salivary conductivity as a biomarker can be a potential method for screening diabetes, with a prediction model having adequate diagnostic accuracy and sensitivity. In conclusion, using a non-invasive, easy-to-use, and portable device to measure salivary conductivity has the possibility to serve as an alternative method for monitoring blood glucose levels, and could be a valuable tool for diabetes screening in the future.

## Figures and Tables

**Table 1:** Baseline characteristics of participants stratified by Diabetes (*n* = 395).

	All (N = 395)	DM (N = 46)	Non-DM (N = 349)	*p*-Value
Salivary conductivity, ms/cm	5.58 ± 1.61	6.29 ± 1.58	5.48 ± 1.59	<0.01
Demographics				
Age, years	51.78 ± 11.31	56.96 ± 6.78	51.10 ± 11.61	<0.01
Gender (male), *n* (%)	126 (31.9)	15 (32.7)	111 (31.8)	0.91
Body weight, kg	64.43 ± 12.53	70.41 ± 12.90	63.65 ± 12.29	<0.01
Body height, cm	160.65 ± 7.84	160.66 ± 7.94	160.65 ± 7.83	0.91
Body mass index, kg/m^2^	24.90 ± 4.15	27.19 ± 4.01	24.60 ± 4.08	<0.01
Systolic blood pressure, mmHg	128.36 ± 21.15	138.87 ± 24.84	126.97 ± 20.26	<0.01
Diastolic blood pressure, mmHg	78.72 ± 12.61	82.24 ± 12.61	78.26 ± 12.55	0.04
Comorbid conditions, *n* (%) ^@^				
Known history of DM	46 (11.6)	26 (56.5)	20 (5.7)	<0.01
Hypertension	95 (24.1)	19 (41.3)	76 (21.8)	<0.01
Dyslipidemia	47 (11.9)	10 (21.7)	37 (10.6)	0.03
Gout	11 (2.8)	1 (2.2)	10 (2.9)	1.00
Laboratory parameters				
BUN, mg/dL	13.97 ± 4.11	14.85 ± 4.65	13.86 ± 4.03	0.11
Creatinine, mg/dL	0.74 ± 0.16	0.73 ± 0.16	0.74 ± 0.16	0.44
eGFR, mL/min/1.73 m^2^	101.54 ± 21.18	101.97 ± 22.20	101.49 ± 21.08	0.86
Serum osmolality, mOsm/kgH_2_O	291.41 ± 6.63	294.91 ± 5.94	290.95 ± 6.58	<0.01
Fasting glucose, mg/dL	108.40 ± 35.69	170.15 ± 56.35	100.26 ± 21.47	<0.01
Hemoglobin A1c, %	5.88 ± 0.93	7.80 ± 1.51	5.63 ± 0.35	<0.01

Values are expressed as mean ± standard deviation or number (percentage). @ The information on comorbid conditions was obtained by questionnaires. Abbreviations: BUN, blood urea nitrogen; eGFR, estimated glomerular filtration rate; DM, diabetes mellitus.

**Table 2:** Population characteristics of low and high salivary conductivity groups.

	Low Salivary Conductivity Group * (N = 251)	High Salivary Conductivity Group (N = 144)	*p*-Value
Salivary conductivity, ms/cm	4.57 ± 0.81	7.33 ± 1.06	<0.01 ^#^
Demographics			
Age, years	50.57 ± 11.35	53.90 ± 10.95	<0.01 ^#^
Gender (male), *n* (%)	79 (31.5)	47 (32.6)	0.81
Body weight, kg	62.47 ± 11.32	67.85 ± 13.79	<0.01 ^#^
Body height, cm	160.37 ± 7.44	161.15 ± 8.49	0.61
Body mass index, kg/m^2^	24.23 ± 3.67	26.07 ± 4.65	<0.01 ^#^
Systolic blood pressure, mmHg	125.81 ± 20.06	132.80 ± 22.32	<0.01 ^#^
Diastolic blood pressure, mmHg	77.76 ± 12.46	80.40 ± 12.73	0.05 ^#^
Comorbid conditions, *n* (%)			
Known history of DM	17 (6.8)	29 (20.1)	<0.01 ^#^
Hypertension	51 (20.3)	44 (30.6)	0.03 ^#^
Dyslipidemia	23 (9.2)	24 (16.7)	0.03 ^#^
Gout	7 (2.8)	4 (2.8)	1.00
Laboratory parameters			
BUN, mg/dL	13.71 ± 4.20	14.44 ± 3.93	0.02 ^#^
Creatinine, mg/dL	0.73 ± 0.16	0.76 ± 0.16	0.05 ^#^
eGFR, mL/min/1.73 m^2^	103.20 ± 20.48	98.65 ± 22.14	0.01 ^#^
Serum osmolality, mOsm/kgH_2_O	291.09 ± 6.83	291.97 ± 6.23	0.12
Fasting glucose, mg/dL	105.02 ± 33.83	114.30 ± 38.12	<0.01 ^#^
Hemoglobin A1c, %	5.77 ± 0.88	6.08 ± 1.00	<0.01 ^#^

Values are expressed as mean ± standard deviation or number (percentage). * Study populations were stratified into low and high groups according to the cutoff value of salivary conductivity (5.987 ms/cm). ^#^ indicates *p* value < 0.05. Abbreviations: BUN, blood urea nitrogen; eGFR, estimated glomerular filtration rate; DM, diabetes mellitus.

**Table 3:** The crude and adjusted odds ratios for the association between salivary conductivity and the risk of diabetes.

Model	Odds Ratio	(95% CI)	*p*-Value
Crude	3.82	1.44–5.56	<0.01
Model 1 *	3.35	1.74–6.46	<0.01
Model 2 ^#^	2.69	1.36–5.32	<0.01

Abbreviations: CI, confidence interval. * Model 1 was adjusted for age and gender. ^#^ Model 2 was adjusted for age, gender, body mass index, systolic blood pressure, diastolic blood pressure, and estimated glomerular filtration rate.

**Table 4:** Crude and adjusted odds ratios for the association between the clinical parameters and the risk of diabetes.

Parameters	Unadjusted		Adjusted			
	Crude		Model 1 *		Model 2 ^#^	
	Odds Ratio	*p*-Value	Odds Ratio	*p*-Value	Odds Ratio	*p*-Value
Age	1.06	<0.01	1.05	<0.01	1.05	0.02
Gender	1.04	0.91	1.02	0.95	0.98	0.97
BMI	1.15	<0.01			1.11	<0.01
SBP	1.03	<0.01			1.02	0.05
DBP	1.02	0.05			0.99	0.46
eGFR	1.00	0.88			1.01	0.22
Salivary conductivity	3.82	<0.01	3.35	<0.01	2.65	<0.01

Abbreviations: BMI, body mass index; DBP, diastolic blood pressure; eGFR, estimated glomerular filtration rate; SBP, systolic blood pressure. * Model 1 was adjusted for age, gender, and salivary conductivity. ^#^ Model 2 was adjusted for age, gender, body mass index, systolic blood pressure, diastolic blood pressure, estimated glomerular filtration rate, and salivary conductivity.

## Data Availability

The data presented in this study are available on request from the corresponding author. The data are not publicly available due to privacy.

## Supplementary Materials

The following supporting information can be downloaded at: https://www.mdpi.com/article/10.3390/bios13070702/s1, Figure S1: Distribution plots of salivary conductivity among different groups; Table S1: Normality tests of the sample data in Table 1 using Kolmogorov-Smirnov method; Table S2: Normality tests of the sample data in Table 2 using Kolmogorov-Smirnov method; Table S3: Baseline characteristics of male participants stratified by Diabetes (n = 126); Table S4: Baseline characteristics of female participants stratified by Diabetes (n = 269) Table S5 Comparison of demographics and laboratory parameters between diabetes and non-diabetes after propensity-score matching (n = 230).
