# Supplementary material for: A Novel Salivary Sensor with Integrated Au Electrodes and Conductivity Meters for Screening of Diabetes
Source: Biosensors (Basel). 2023 Jul 2;13(7):702. doi: 10.3390/bios13070702 (PMC10377178; doi:10.3390/bios13070702)
Supplement: Supplementary file 1 [file biosensors-13-00702-s001.zip › biosensors-2418143-supplementary.pdf]

Supplemental Materials

C-W Lin, Y-H Tsai and Y-S Peng, et al. A Novel Salivary Sensor with Integrated Au Electrodes and Conductivity Meters for Screening of Diabetes

Content

Table S1. Normality tests of the sample data in Table 1 using Kolmogorov-Smirnov method

Table S2. Normality tests of the sample data in Table 2 using Kolmogorov-Smirnov method

Table S3. Baseline characteristics of male participants stratified by Diabetes (n=126)

Table S4. Baseline characteristics of female participants stratified by Diabetes (n=269)

Table S5. Comparison of demographics and laboratory parameters between diabetes and non-diabetes after propensity-score matching (n = 230).

Figure S1. Distribution plots of salivary conductivity among different groups

Table S1. Normality tests of the sample data in Table 1 using Kolmogorov-Smirnov method

	DM (N=46)	P value	Non-DM (N=349)	P value
**Salivary conductivity, ms/cm**	6.29±1.58	0.64	5.48±1.59	<0.01
**Demographics**				
Age, years	56.96±6.78	<0.01	51.10±11.61	<0.01
Gender (male), n (%)	15 (32.7)	NA	111 (31.8)	NA
Body weight, kg	70.41±12.90	0.57	63.65±12.29	<0.01
Body height, cm	160.66±7.94	0.64	160.65±7.83	<0.01
Body mass index, kg/m^2	27.19±4.01	0.35	24.60±4.08	<0.01
Systolic blood pressure, mmHg	138.87±24.84	0.23	126.97±20.26	<0.01
Diastolic blood pressure, mmHg	82.24±12.61	0.79	78.26±12.55	0.09
**Comorbid conditions, n (%)** @				
Known history of DM	26 (56.5)	NA	20 (5.7)	NA
Hypertension	19 (41.3)	NA	76 (21.8)	NA
Dyslipidemia	10 (21.7)	NA	37 (10.6)	NA
Gout	1 (2.2)	NA	10 (2.9)	NA
**Laboratory parameters**				
BUN, mg/dL	14.85±4.65	0.11	13.86±4.03	<0.01
Creatinine, mg/dL	0.73±0.16	0.01	0.74±0.16	<0.01
eGFR, mL/min/1.73 m^2	101.97±22.20	0.18	101.49±21.08	<0.01
Serum osmolality, mOsm/kgH_2O	294.91±5.94	<0.01	290.95±6.58	<0.01
Fasting glucose, mg/dL	170.15±56.35	<0.01	100.26±21.47	<0.01
Hemoglobin A1c, %	7.80±1.51	<0.01	5.63±0.35	<0.01

Values are expressed as mean ± standard deviation or number (percentage). @ The information on comorbid conditions was obtained by questionnaires. Abbreviations: BUN, blood urea nitrogen; eGFR, estimated glomerular filtration rate; DM, diabetes mellitus; NA, not applicable. P value < 0.05 indicates that the data is not normally distributed.

Table S2. Normality tests of the sample data in Table 2 using Kolmogorov-Smirnov method

	Low salivary conductivity group (N=251)	P value	High salivary conductivity group (N=144)	P value
**Salivary conductivity, ms/cm**	4.57±0.81	<0.01	7.33±1.06	<0.01
**Demographics**				
Age, years	50.57±11.35	<0.01	53.90±10.95	<0.01
Gender (male), n (%)	79 (31.5)	NA	47 (32.6)	NA
Body weight, kg	62.47±11.32	<0.01	67.85±13.79	0.20
Body height, cm	160.37±7.44	0.22	161.15±8.49	<0.01
Body mass index, kg/m^2	24.23±3.67	<0.01	26.07±4.65	<0.01
Systolic blood pressure, mmHg	125.81±20.06	<0.01	132.80±22.32	<0.01
Diastolic blood pressure, mmHg	77.76±12.46	0.17	80.40±12.73	0.33
**Comorbid conditions, n (%) @**				
Known history of DM	17 (6.8)	NA	29 (20.1)	NA
Hypertension	51 (20.3)	NA	44 (30.6)	NA
Dyslipidemia	23 (9.2)	NA	24 (16.7)	NA
Gout	7 (2.8)	NA	4 (2.8)	NA
**Laboratory parameters**				
BUN, mg/dL	13.71±4.20	<0.01	14.44±3.93	<0.01
Creatinine, mg/dL	0.73±0.16	<0.01	0.76±0.16	<0.01
eGFR, mL/min/1.73 m^2	103.20±20.48	<0.01	98.65±22.14	<0.01
Serum osmolality, mOsm/kgH_2O	291.09±6.83	<0.01	291.97±6.23	<0.01
Fasting glucose, mg/dL	105.02±33.83	<0.01	114.30±38.12	<0.01
Hemoglobin A1c, %	5.77±0.88	<0.01	6.08±1.00	<0.01

Values are expressed as mean ± standard deviation or number (percentage). @ The information on comorbid conditions was obtained by questionnaires. Abbreviations: BUN, blood urea nitrogen; eGFR, estimated glomerular filtration rate; DM, diabetes mellitus; NA, not applicable. P value < 0.05 indicates that the data is not normally distributed.

Table S3. Baseline characteristics of male participants stratified by Diabetes (n=126)

	Male (N=126)	DM (N=15)	Non-DM (N=111)	P value
**Salivary conductivity, ms/cm**	5.65±1.56	6.48±1.31	5.53±1.56	0.02
**Demographics**				
Age, years	51.76±11.78	54.87±7.26	51.34±12.23	0.12
Body weight, kg	72.07±12.61	77.80±11.60	71.30±12.59	0.06
Body height, cm	168.37±6.47	168.39±6.09	168.37±6.55	0.99
Body mass index, kg/m^2	25.37±3.81	27.39±3.28	25.09±3.81	0.02
Systolic blood pressure, mmHg	132.79±18.65	142.27±18.51	131.51±18.37	0.05
Diastolic blood pressure, mmHg	81.47±12.72	86.73±12.94	80.76±12.58	0.11
**Comorbid conditions, n (%) @**				
Known history of DM	16 (12.7)	5 (33.3)	11 (9.9)	<0.01
Hypertension	35 (27.8)	5 (33.3)	30 (27.0)	0.61
Dyslipidemia	14 (11.1)	1 (6.7)	13 (11.7)	0.49
Gout	9 (7.1)	1 (6.7)	8 (7.2)	0.94
**Laboratory parameters**				
BUN, mg/dL	14.29±3.78	14.61±4.39	14.25±3.71	0.76
Creatinine, mg/dL	0.90±0.15	0.84±0.19	0.90±0.14	0.24
eGFR, mL/min/1.73 m^2	98.24±21.30	106.84±27.12	97.08±20.26	0.20
Serum osmolality, mOsm/kgH_2O	292.04±6.94	295.47±4.73	291.58±7.08	<0.01
Fasting glucose, mg/dL	107.43±33.81	158.20±48.94	100.57±24.39	<0.01
Hemoglobin A1c, %	5.88±0.72	7.38±0.94	5.68±0.35	<0.01

Values are expressed as mean ± standard deviation or number (percentage). @ The information on comorbid conditions was obtained by questionnaires. Abbreviations: BUN, blood urea nitrogen; eGFR, estimated glomerular filtration rate; DM, diabetes mellitus.

Table S4. Baseline characteristics of female participants stratified by Diabetes (n=269)

	Female (N=269)	DM (N=31)	Non-DM (N=238)	P value
**Salivary conductivity, ms/cm**	5.55±1.64	6.29±1.58	5.48±1.59	<0.01
**Demographics**				
Age, years	51.79±11.10	57.97±6.42	50.99±11.33	<0.01
Body weight, kg	60.85±10.79	66.84±12.09	60.08±10.39	<0.01
Body height, cm	157.04±5.44	156.92±5.74	157.05±5.41	0.91
Body mass index, kg/m^2	24.68±4.29	27.09±4.36	24.37±4.18	<0.01
Systolic blood pressure, mmHg	126.28±21.96	137.23±27.51	124.86±20.78	0.02
Diastolic blood pressure, mmHg	77.44±12.37	80.06±12.06	77.10±12.39	0.21
**Comorbid conditions, n (%) [@]**				
Known history of DM	30 (11.2)	21 (67.7)	9 (3.4)	<0.01
Hypertension	60 (22.3)	13 (41.9)	47 (19.7)	<0.01
Dyslipidemia	33 (12.3)	9 (29.0)	24 (10.1)	<0.01
Gout	2 (0.7)	0 (0)	2 (0.8)	-
**Laboratory parameters**				
BUN, mg/dL	13.82±4.26	14.97±4.84	13.68±4.17	0.16
Creatinine, mg/dL	0.68±0.11	0.67±0.12	0.67±0.10	0.88
eGFR, mL/min/1.73 m^2	103.09±20.99	99.61±19.45	103.54±21.18	0.30
Serum osmolality, mOsm/kgH_2O	291.12±6.47	294.65±6.50	290.66±6.33	<0.01
Fasting glucose, mg/dL	108.86±36.59	175.94±59.49	100.12±20.02	<0.01
Hemoglobin A1c, %	5.88±1.01	8.01±1.70	5.61±0.35	<0.01

Values are expressed as mean ± standard deviation or number (percentage). @ The information on comorbid conditions was obtained by questionnaires. Abbreviations: BUN, blood urea nitrogen; eGFR, estimated glomerular filtration rate; DM, diabetes mellitis

Table S5. Comparison of demographics and laboratory parameters between diabetes and non-diabetes after propensity-score matching (n = 230).

	DM (N=46)	Non-DM (N=184)	P value
**Salivary conductivity, ms/cm**	6.29±1.58	5.46±1.49	<0.01
**Demographics**			
Age, years	56.96±6.78	57.95±5.52	0.30
Gender (male), n (%)	15 (32.7)	61 (33.2)	0.94
Body weight, kg	70.41±12.90	65.89±11.66	0.02
Body height, cm	160.66±7.94	159.67±7.67	0.43
Body mass index, kg/m^2	27.19±4.01	25.78±3.87	0.03
Systolic blood pressure, mmHg	138.87±24.84	135.24±19.57	0.36
Diastolic blood pressure, mmHg	82.24±12.61	81.26±11.77	0.62
**Comorbid conditions, n (%)**			
Known history of DM	26 (56.5)	16 (8.7)	<0.01
Hypertension	19 (41.3)	68 (37.0)	0.79
Ischemic heart disease/ Stroke	0 (0)	1 (0.5)	1.00
Dyslipidemia	10 (21.7)	30 (16.3)	0.40
Gout	1 (2.2)	8 (4.3)	0.47
**Laboratory parameters**			
BUN, mg/dL	14.85±4.65	14.46±4.02	0.57
Creatinine, mg/dL	0.73±0.16	0.74±0.16	0.49
BUN/Cr ratio	21.04±7.07	20.05±6.39	0.36
eGFR, mL/min/1.73 m^2	101.97±22.20	98.47±20.44	0.31
Serum osmolality, mOsm/kgH_2O	294.91±5.94	292.17±6.54	0.01
Fasting glucose, mg/dL	170.15±56.35	103.71±21.98	<0.01
Hemoglobin A1c, %	7.80±1.51	5.73±0.33	<0.01
ALT, U/L	30.83±17.49	24.05±13.68	0.02
Triglyceride, mg/dL	181.30±114.53	125.73±71.83	<0.01
Total cholesterol, mg/dL	195.39±40.56	201.78±38.29	0.32
LDL-C, mg/dL	111.67±35.11	120.85±35.62	0.12
HDL-C, mg/dL	48.74±13.28	55.89±14.43	<0.01

Values are expressed as mean ± standard deviation or number (percentage). @ The information on comorbid conditions was obtained by questionnaires. Abbreviations: BUN, blood urea nitrogen; eGFR, estimated glomerular filtration rate; DM, diabetes mellitis

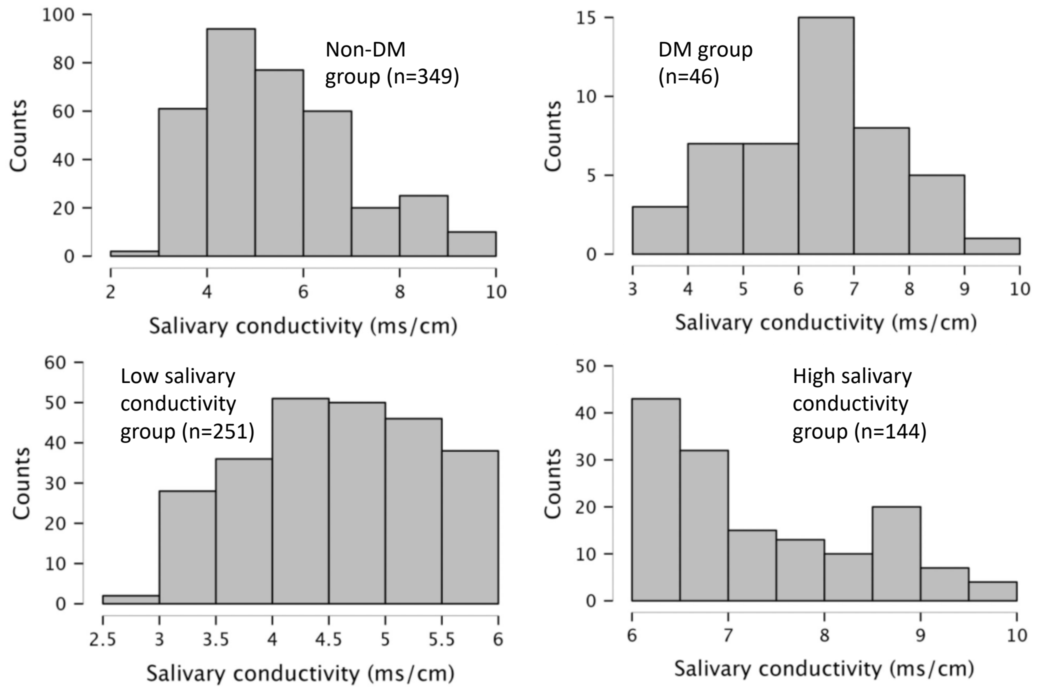

Figure S1. Distribution plots of salivary conductivity among different groups
